# Optical Manipulation of Liquids by Thermal Marangoni Flow along the Air–Water Interfaces of a Superhydrophobic Surface

**DOI:** 10.1021/acs.langmuir.1c00539

**Published:** 2021-07-14

**Authors:** Aiting Gao, Hans-Jürgen Butt, Werner Steffen, Clarissa Schönecker

**Affiliations:** † Max Planck Institute for Polymer Research, Ackermannweg 10, D-55128 Mainz, Germany; ‡ TU Kaiserslautern, Group for Micro Fluid Mechanics, Gottlieb-Daimler-Straße 49, 67663 Kaiserslautern, Germany

## Abstract

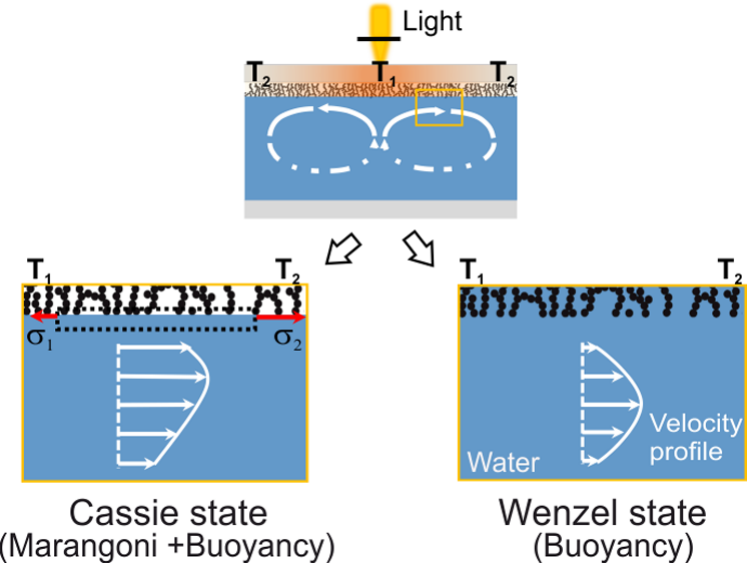

The control of liquid motion on the micrometer scale is important for many liquid transport and biomedical applications. An efficient way to trigger liquid motion is by introducing surface tension gradients on free liquid interfaces leading to the Marangoni effect. However, a pronounced Marangoni-driven flow generally only occurs at a liquid–air or liquid–liquid interface but not at solid–liquid interfaces. Using superhydrophobic surfaces, the liquid phase stays in the Cassie state (where liquid is only in contact with the tips of the rough surface structure and air is enclosed in the indentations of the roughness) and hence provides the necessary liquid–air interface to trigger evident Marangoni flows. We use light to asymmetrically heat this interface and thereby control liquid motion near superhydrophobic surfaces. By laser scanning confocal microscopy, we determine the velocity distribution evolving through optical excitation. We show that Marangoni flow can be induced optically at structured, air-entrapping superhydrophobic surfaces. Furthermore, by comparison with numerical modeling, we demonstrate that in addition to the Marangoni flow, buoyancy-driven flow occurs. This effect has so far been neglected in similar approaches and models of thermocapillary driven flow at superhydrophobic surfaces. Our work yields insight into the physics of Marangoni flow and can help in designing new contactless, light-driven liquid transport systems, e.g., for liquid pumping or in microfluidic devices.

## Introduction

Controlled liquid transport, especially guiding liquid flows on the microscale, is crucial for numerous applications, such as microfluidics for chemical or biochemical analysis or inkjet printing techniques. However, traditional pressure-driven liquid transport becomes difficult at small scales. For instance, driving liquid flow through capillaries becomes less and less energy efficient the smaller the capillaries are. This is because, according to the law of Hagen-Poisseuille, the flow at a given pressure difference scales with the fourth power of the radius.

With decreasing system size, the surface to volume ratio of the fluid increases significantly and correspondingly controls the fluid flow. Hence, interfaces provide an excellent platform for manipulating flows at small scales. When a surface stress is generated at a fluid–fluid interface, it spontaneously induces a deformation or dynamic motion. For instance, surface-driven flows are induced by a surface tension gradient at the fluid–fluid interface, which is also known as the Marangoni effect.^1^ It is generated when the fluid interface is subjected to a thermal or concentration gradient. The Marangoni effect has been reported to induce directional/convective flows in the liquid phase,^2−5^ to guide droplets or floating objects at fluid interfaces,^6−9^ and to determine various spreading dynamics phenomena, e.g., fingering instabilities,^10,11^ “Marangoni bursting”.^12^ In addition to these observations, the Marangoni effect has been reported as a promising approach for the actuation of droplets or liquid films in microfluidic systems,^10,13−20^ e.g., for directional transport of droplets on a nonuniformly heated solid surface^16,17,21^ or when a droplet is immersed in another immiscible liquid which is imposed with a temperature gradient.^22−27^

However, apart from systems such as droplets or films, where free fluid–fluid interfaces are immanently present, the Marangoni effect has been rarely reported to trigger continuous flows in confined liquid systems, e.g., pure liquid flows in enclosed microfluidic devices such as channels, due to the lack of free liquid interfaces. The liquid being in contact with the solid wall, or the liquid–solid interface, is normally considered as a no-slip boundary (i.e., the velocity is zero). To overcome this problem, the use of a structured, air-entrapping superhydrophobic surface has been proposed to introduce a liquid–fluid interface and thereby a thermocapillary-driven Marangoni flow close to a solid interface.^38^ Superhydrophobic surfaces have attracted intensive attention due to their liquid repellency as well as drag reduction properties.^28−35^ The liquid phase, which is in contact with these surfaces, cannot penetrate the features of the surface roughness, which leads to an air phase remaining in the valleys of the surface roughness (or the so-called Cassie state).^36,37^ This provides a discontinuous liquid–air interface close to the solid surface. Baier et al. have theoretically shown that a temperature gradient imposed along a superhydrophobic surface can induce directional thermocapillary-driven flows in the liquid phase along the direction the temperature decreases.^38^ Later, Amador et al. demonstrated this principle experimentally, obtaining a thermocapillary flow with a speed of 10–50 μm/s in a submillimeter-wide channel with superhydrophobic sidewalls and an imposed temperature gradient of 2 K/cm.^39^ These two works have shown the possibility of triggering liquid motion by the thermocapillary effect.

Beyond the basic principle, a smart and precise control of thermocapillary flows over superhydrophobic surfaces has not yet been realized. An optothermal approach shows the possibility to achieve this goal without any complicated heating and cooling setups. While heating and cooling elements are typically fixed in place, an activation by light could be much more flexible, e.g., by moving a light beam along a superhydrophobic surface, as well as by varying the light intensity and illuminated area and, hence, the temperature gradient. In the experimental work of Amador et al.,^39^ it was shown that a flow could be created by coating half of the channel with black ink. However, no precise control of the temperature gradients have been realized. Furthermore, the physical details of this kind of flow have not yet been investigated.

At small dimensions, the thermocapillary flow is typically considered the main source of fluid propulsion. Other thermally induced physical effects, especially temperature-induced change in the liquid density (buoyancy force), are usually neglected. However, this may not correct for thermocapillary flows over superhydrophobic surfaces. Because of the small slip length of the superhydrophobic surface (usually a few micrometer),^30,40^ the corresponding thermocapillary flow is much weaker than on a free liquid interface. Similar to the case of buoyant-thermocapillary instabilities in a liquid phase with a free upper interface^41−45^ or in binary droplets,^46−49^ we may need to consider both thermal effects. However, to the best of our knowledge, no theoretical models and experiments on thermocapillary flows at superhydrophobic surfaces including buoyancy have so far been reported. In this study, we want to clarify this issue to better understand the flow mechanism over superhydrophobic surfaces.

**Figure 1:**
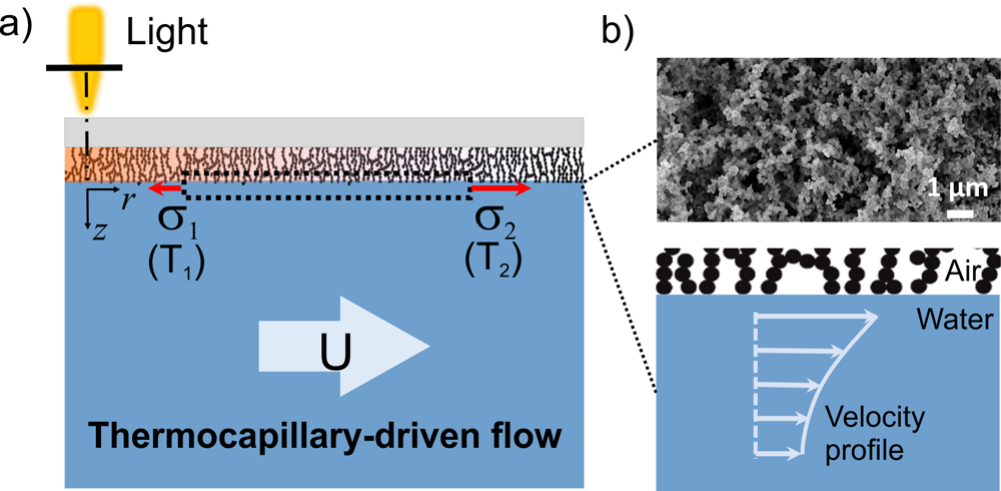
Concept of optothermally induced thermocapillary flow in a liquid in contact with a superhydrophobic surface in the Cassie state. (a) Schematic of the thermocapillary driven flow induced by nonuniform light irradiation on the surface covered with soot (at the top). Due to the photothermal effect, the substrate is heated and conducts the heat to the liquid. The nonuniform temperature distribution at the liquid interface causes a gradient in the surface tension σ at the liquid–air interface ( or ⟨*∂T*⟩_*r*_ in the *r* direction), which leads to a surface flow (*U*). (b) Microstructures of the soot surface imaged by SEM and schematic of the discontinuous liquid–air interface at the superhydrophobic surface.

Therefore, we study an optical approach to manipulate liquid motion by exploiting the thermocapillary effect along the air–water interfaces of a rough superhydrophobic surface. We constructed a superhydrophobic surface with a photothermal material. Nonuniform heating of the surface under illumination with light allows the generation of a nonuniform temperature distribution at the surface and in the liquid phase. This distribution leads to the generation of surface tension gradients at the liquid–air interface near the superhydrophobic surface driving a thermocapillary flow (Figure 1). Furthermore, density gradients in the liquid phase may occur, which can additionally trigger buoyancy-driven flows. Our experimental approach of having the heated surface at the top allows us to distinguish between these two effects. Combining the experimental observations with theoretical simulations enables us to study the underlying physics. This work provides a basic understanding of thermally triggered flows on superhydrophobic surfaces.

## Experimental Methods

A superhydrophobic surface fabricated from photothermal material, was placed on top of a water reservoir in a circular μ-Slide chamber (with height *H* ∼ 1 mm and diameter *D* ∼ 21 mm, Figure S1). A focused laser (diode laser, CW mode, wavelength *λ*_exe_ = 488 nm, spot size diameter ⌀ ∼ 350 μm, and power *Q*_laser_ = 10 mW at the sample, Cobolt 06–01 series) was applied to irradiate the superhydrophobic surface from the top. An infrared camera, with a 0.5× converter lens installed ahead (VarioCAM HD head 680s, IR 1.0/60 LW JENOPTIK, InfraTec, Germany), was used to characterize the temperature of the upper superhydrophobic surface and the bottom surface of the chamber with a time resolution of 25 frames/second.

The liquid motion in the water phase was recorded by a confocal microscope (Leica LCS-SP8) with a water objective (Leica HCX IRAPO L 25× 0.95 W, free working distance: 2.5 mm). Two kinds of fluorescent polystyrene particles were used as tracers: micrometer-sized PS beads (diameter 2.5 μm, λ_exe_ = 532 nm, concentration ∼ 1.2 × 10^6^ /mL, Life technologies, USA) and submicrometer-sized PS beads (diameter 200 nm, dyed with Nile red, λ_exe_ = 488 nm, concentration ∼10^6^ /mL, synthesized in our lab by soap-less emulsion polymerization^50,51^).

The particle transport processes were recorded by scanning perpendicular (xzt mode) or parallel (xyt mode) to the liquid layer’s top surface. We repeated the scans in time (xytz mode, *Z* steps, the axial step interval *Z*_step-size_ = 3 μm) to record the motion of the particles in time. This was repeated for different liquid layer depths. 500 images were captured at 15 fps. The captured image size was 512 × 512 pixels, leading to a spatial resolution of approximately 0.73 μm. Each experiment was repeated at least three times before statistical analysis.

In our study, we used three different solid surfaces: (1) a superhydrophobic soot surface (contact angle θ > 160°, contact angle hysteresis θ_CAH_ < 5°), (2) a superhydrophilic soot surface (θ < 10°), and (3) a superhydrophobic pillar surface (θ > 150°°, θ_CAH_ < 10°°). In all three cases, carbon black (soot) was employed to enhance the photothermal effect and heat conduction in the solid.

The superhydrophobic and superhydrophilic soot surfaces were prepared as reported by Deng et al.^52^ (see the Supporting Information for details of the fabrication methods of the different surfaces). An *h* ≈ 30 μm thick soot layer was generated from candle wax on the glass slide. Then, a silica shell was deposited through chemical vapor deposition (CVD) and an additional solution approach. Within this two-step Stöber reaction process, tetraethyl orthosilicate (with ammonia as a catalyst) produced silicon oxide on the soot surface.^13^ These surfaces were either left as-is, which were the hydrophilic soot-based surfaces, or additionally fluorinated with (tridecafluoro-1,1,2,2-tetrahydrooctyl)-1-trichlorosilane (97%, Sigma-Aldrich) to yield superhydrophobic surfaces. The fluorination was done in a sealed desiccator vacuumed to <200 mbar.

The superhydrophobic pillar surfaces were cast in silicone elastomer from a SU-8 round pillar mold with a round lattice of cylindrical micropillars (pillar diameter *a* ∼ 10 μm, space between pillars *d* ∼ 20 μm, and height *h*∼ 30 μm). We added black carbon particles (with average diameter ∼42 nm, Thermo Fisher) to the silicone elastomer (Sylgard 184, Dow Corning, with a mass weight ratio of 500:1) to obtain solid properties as close to the soot-coated surfaces as possible. After curing (6h at 60 °C), a black silicone surface was obtained. Additionally, it was decorated with μm-sized PS particles by depositing a dense and well-ordered PS particle film via a Langmuir–Blodgett-like (LB-like) technique.^14,15^ Finally, the surface was fluorinated (see above) to stabilize the Cassie state.

The soot-based superhydrophobic surface (Figure 1b) was the main surface to examine the thermocapillary-driven flows in the liquid phase. The soot-based superhydrophilic surfaces served for comparison. Its hydrophilicity leads to wetting of the surface in the Wenzel state. Without air entrapped in the surface roughness features, there is no thermocapillary-driven flow possible on this surface, while the surface geometry and other surface properties are equivalent to the soot-based superhydrophobic surface. To compare the role of the surface morphology, i.e., of the effective slip length, we used the pillar surfaces. The micrometer-sized pillars provide a larger slip length than the soot surfaces, which are structured on the nanometer scale.

## Numerical Model

To compare and identify the different mechanisms occurring in the experiments, we built a numerical model of the flow expected in the experimental setup. The stationary mass and momentum conservation equations read:
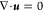
1
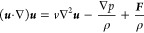
2Here, ***u*** is the velocity field; ρ and *v* are the density and kinetic viscosity of the liquid; *p* is the fluid pressure; and ***F*** is a body force per unit volume acting on the fluid (N/m^3^). The only body force that may have to be considered in this system is due to gravity, which is associated with density differences of the liquid phase. It is expressed as ***F*** = ρ***g***β(*T* – *T*_0_), where β is the thermal expansion coefficient of the liquid phase and ***g*** is the gravitational acceleration. The corresponding heat transport in the system is described by
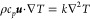
3where *c*_*p*_ and *k* are the specific heat capacity and thermal conductivity. For water at room temperature, *v* ∼ 8.917 × 10^–7^ m^2^/s, β = 2.570 × 10^–4^ K^–1^, *c*_*p*_ = 4.182 kJ/(kg K), and *k* = 0.608 W/(m K).

These equations were solved using the finite element simulation software COMSOL (COMSOL Multiphysics Software 5.4a, Comsol, Inc., Stockholm, Sweden) in an axisymmetric geometry corresponding to the experimental setup (*R* = 20 mm, *Z* = 1 mm). To identify the different mechanisms occurring in the experimental flow measurements, we theoretically defined three generic scenarios: (1) Marangoni stress at the superhydrophobic surface without buoyancy forces in the liquid, (2) no Marangoni stress, but buoyancy forces in the liquid, and (3) Marangoni stress at the superhydrophobic surface and buoyancy forces in the liquid.

Case (1) is the classic case assumed in Baier et al.^38^ and the following theoretical studies. At small dimensions, neglecting gravity forces is a common assumption. None of the existing theoretical models on thermocapillary flows at superhydrophobic surfaces has considered buoyancy.^53−56^ In contrast, it is known for free liquid interfaces that buoyancy may occur.^41,43,44^

Boundary conditions for the fluid are no-slip on all walls except for the superhydrophobic surface. Here, a condition for the Marangoni-induced velocity at a superhydrophobic surface is required. In contrast to the typical Navier slip condition that is employed for plain flow over superhydrophobic surfaces (such as pressure or shear driven flow without thermal or other effects), there is no such general effective boundary condition for Marangoni flow on superhydrophobic surfaces. An explicit expression exists merely for the case of a superhydrophobic surface with longitudinal grooves.^38^ In this case, the velocity component parallel to the surface is , with σ being the surface tension of the liquid, *b* the effective slip length of the superhydrophobic surface, and *x* the coordinate along the grooves. The minus sign stems from the fact that  is typically negative. For differently shaped superhydrophobic surfaces than longitudinal grooves, the mathematical problem to be solved to derive a boundary condition is far more intricate.^56^ If, in lack of an appropriate boundary condition, the above expression is analogously applied to differently shaped superhydrophobic surfaces, e.g., to transverse grooves, it may overestimate the actual velocity, especially at large air interface fractions.

In the present case, the geometry of the superhydrophobic surface is complex and even irregular for the soot-based surface. Hence, there is no boundary condition available. As the closest possible condition, we therefore apply the expression for longitudinal grooves, bearing in mind a possible overestimation of the Marangoni flow. Furthermore, within the boundary condition itself, the effective slip length *b* plays a key role for the magnitude of the Marangoni flow. In the present case, the stochastic surface geometry of the soot-based surfaces does not allow an exact determination of the value of the effective slip length. Hence, the magnitude of the effective slip length is estimated from the formulas for comparable grooved surfaces.^57−60^ For a given air-interface fraction, the effective slip length *b* of a 2-dimensionally or randomly structured surface must be in between those for transverse and longitudinal grooves, since these represent the cases where the flow of the water is most and least obstructed by the no-slip condition at the solid parts of the surface. For the soot-based surfaces^61^ we estimate 0.22 < *b* < 0.45 μm with a characteristic distance of *d* = 1 μm between the particle protrusions and a protrusion diameter of *a* = 200 *n*m. For the pillar-structured surface, 3.2 < *b* < 6.4 μm with a pillar spacing of *d* = 20 μm and a pillar diameter of *a* = 10 μm.

In cases (2) and (3), the buoyancy term ***F*** in eq 1 is activated, while it is neglected for case (1). Although there is no Marangoni stress present in case (2), we account for the presence of the superhydrophobic surface by allowing a slip velocity according to the standard Navier slip boundary condition . More details on the boundary conditions are listed in the **SI** (Figure S7 and Table S1).

## Results and Discussion

### Temperature Distribution and Flow Pattern

**Figure 2:**
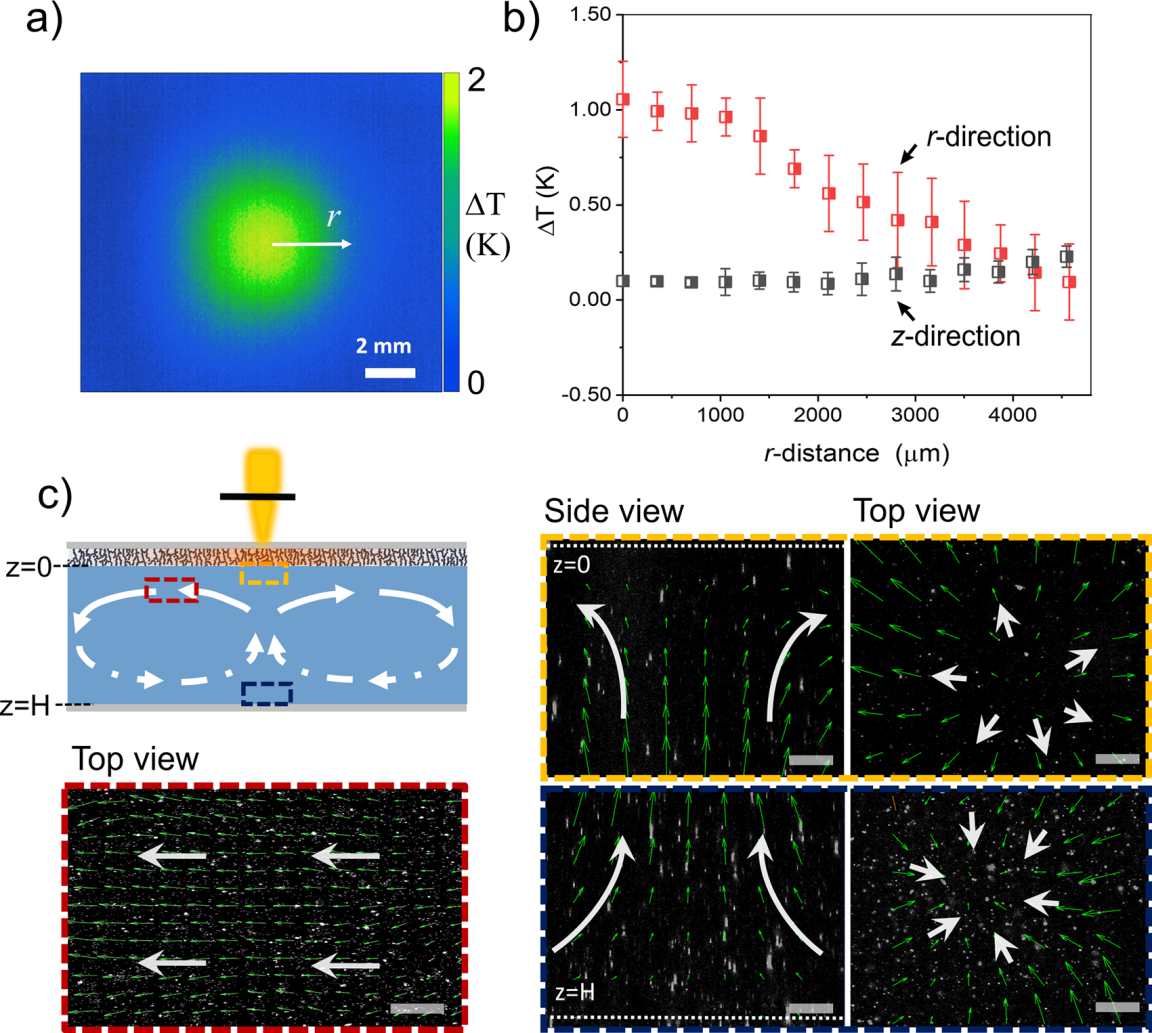
(a) Illustration of the temperature profile near the superhydrophobic surface from the water side, measured by the IR camera. (b) Measured temperature differences on the soot surface: in the *r*-direction, relative to the temperature far away from the heated spot (equals room temperature). In the *z*-direction, differences between top and bottom of the cell at a given distance from the center of the heated spot. (c) Schematic and visualization of the fluid pattern in the liquid layer. Images of fluorescently labeled particles (white dots) superimposed with velocity vectors obtained from PIV analysis (green) in the three regions indicated by colored rectangles. (Scale bars: 50 μm).

The laser beam focused on the soot surface (spot diameter ∼350 μm) creates an axisymmetrically heated spot due to its Gaussian shape in its energy distribution. When the surface is exposed to air, this peak is sharp, corresponding to the focused beam (Figure S4). The presence of water leads to broadening and flattening of the heated spot (Figure 2a). The energy distribution in the laser beam seems then not to play a role.

The heat produced at the soot surface under the laser irradiation is conducted through the liquid, causing only a small temperature difference in the *z*-direction, which points perpendicular to the surface. The temperature distribution in the liquid layer was axisymmetric to the center of the laser beam in the *r*-direction and is shown in Figure 2b. Near the soot surface, a temperature gradient in the *r*-direction of Δ*T*/Δ*r* ∼ 0.25 K/mm was obtained. In the *z*-direction, the difference between the top and bottom surfaces was Δ*T*_*z*_ ∼ 0.1 K. Heat conduction through the water phase is hence an important effect. It both distributes the heat laterally along the substrate and vertically to it. The first one influences the temperature gradient driving the Marangoni flow. The latter locally heats up the water up to a significant depth.

By adding fluorescent particles as tracers, the thermally induced fluid motion in the liquid was recorded by confocal microscopy. Three regions marked by color-coded rectangles in Figure 2c were observed: near the top interface with a horizontal distance from the hot spot center (red, *r* = 500 μm, *z* ∼ 0), near the top interface in the heated spot region (yellow, *r* ∼ 0, *z* ∼ 0) and near the bottom substrate (blue, *r* ∼ 0, *z* ∼ 1000 μm). Near the superhydrophobic surface, directional flows are generated along the superhydrophobic surface due to the directional temperature gradient (Figure 2c). Near the top interface (yellow, *r* ∼ 0, *z* ∼ 0), the tracers move axisymetrically outward in the direction of decreasing temperature. Near the bottom glass surface (*r* ∼ 0, *z* ∼ 1000 μm), the tracers move inward to feed the outward flow at the upper interface. Without the laser switched on, we observed only random (Brownian) motion in our cell.

### Simulation Results

**Figure 3:**
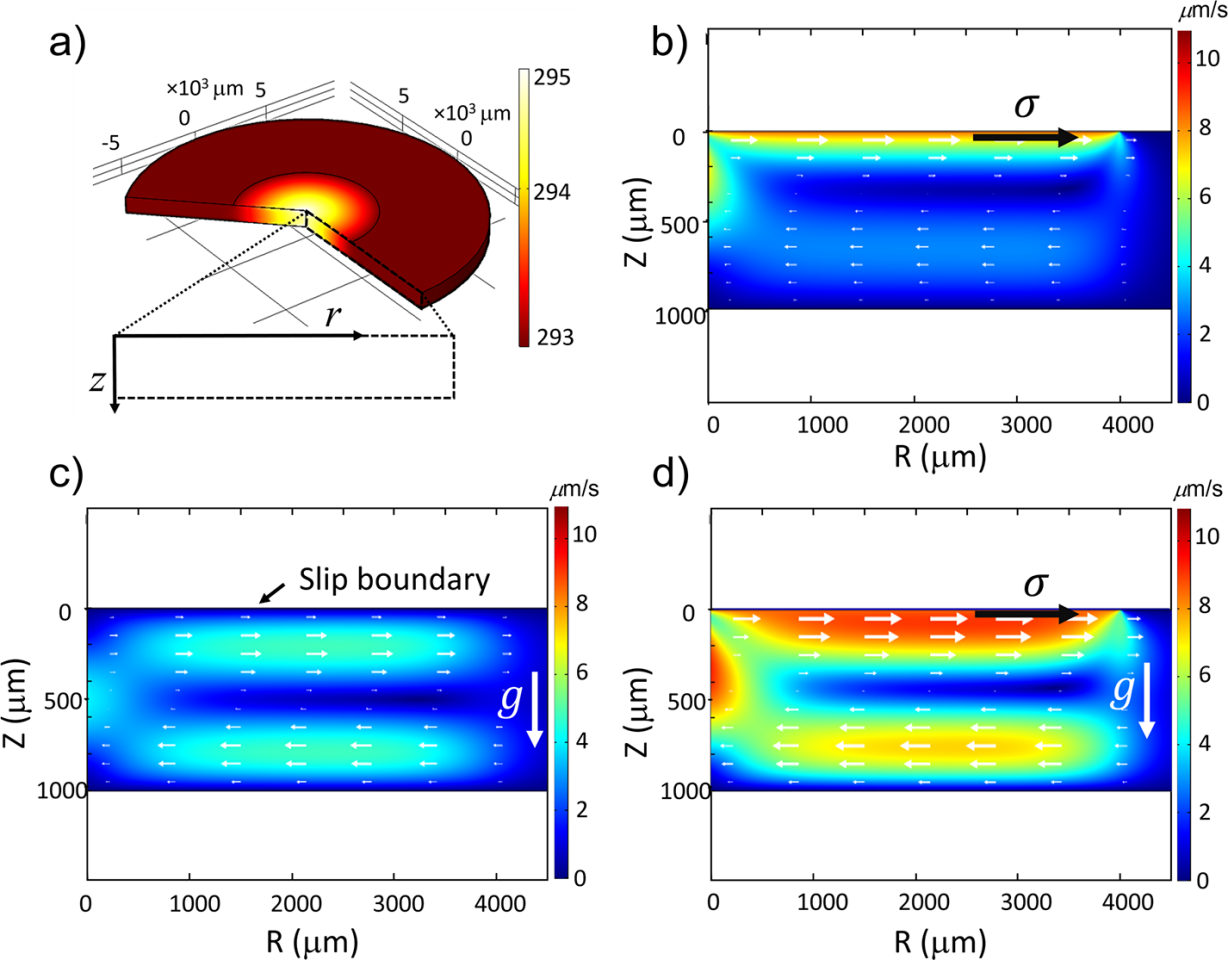
Finite element simulation of the fluid motion under local heating of the superhydrophobic soot surface at the top. (a) Temperature distribution in the simulated system, (b–d) Flow vectors and velocity magnitude for (b) case (1): Marangoni stress at the superhydrophobic surface without buoyancy forces in the liquid; (c) case (2): no Marangoni stress, but buoyancy forces in the liquid; and (d) case (3): Marangoni stress at superhydrophobic surface and buoyancy forces in the liquid. (Related parameters: *b* = 0.22 μm; *∂σ*/*∂T* = −0.155 mN/m K; β = 2.570 × 10^–4^ K; and *T*_0_ = 293.15 K.)

With the use of the experimental temperature distribution as input (Figure 2b for the soot-based superhydrophobic surface, Figure S6 for the other surfaces), finite element simulations were performed for the three cases listed above (Figure 3). The axisymmetric temperature distribution is shown in Figure 3a. In all three cases, axisymmetric toroidal eddies are generated in the liquid phase, which however differ in their velocity distribution. For pure Marangoni flow without buoyancy [case (1), Figure 3b], the flow is concentrated close to the superhydrophobic surface. The highest velocity is at the boundary, which drags the rest of the liquid along. Because the system is closed, there is a backflow through the lower part of the water reservoir. For pure buoyancy [case (2), Figure 3c], the convective rolls are more equally distributed between the upper and lower part of the reservoir. The highest velocity in the upper part is at about 1/4 of the height of the reservoir. The effect of the slip boundary condition at the superhydrophobic surface is only small because the slip length of the soot surface is small. Case (3) (Figure 3d) is a combination of the two previous ones with both the Marangoni stress and the buoyancy causing an outward flow in the upper part of the reservoir. The velocity distribution is nearly a superposition of the two other cases. Since the velocities in the two individual cases are of a similar order of magnitude, the maximum velocity is obtained at a position in between the superhydrophobic boundary and the position for pure buoyancy. If the strength of either Marangoni stress or buoyancy is modified, the position of the maximum velocity also shifts accordingly. In the following, we will use these 3 types of flow to identify the processes occurring in the experiment.

### Velocity Distribution and Mechanisms

**Figure 4:**
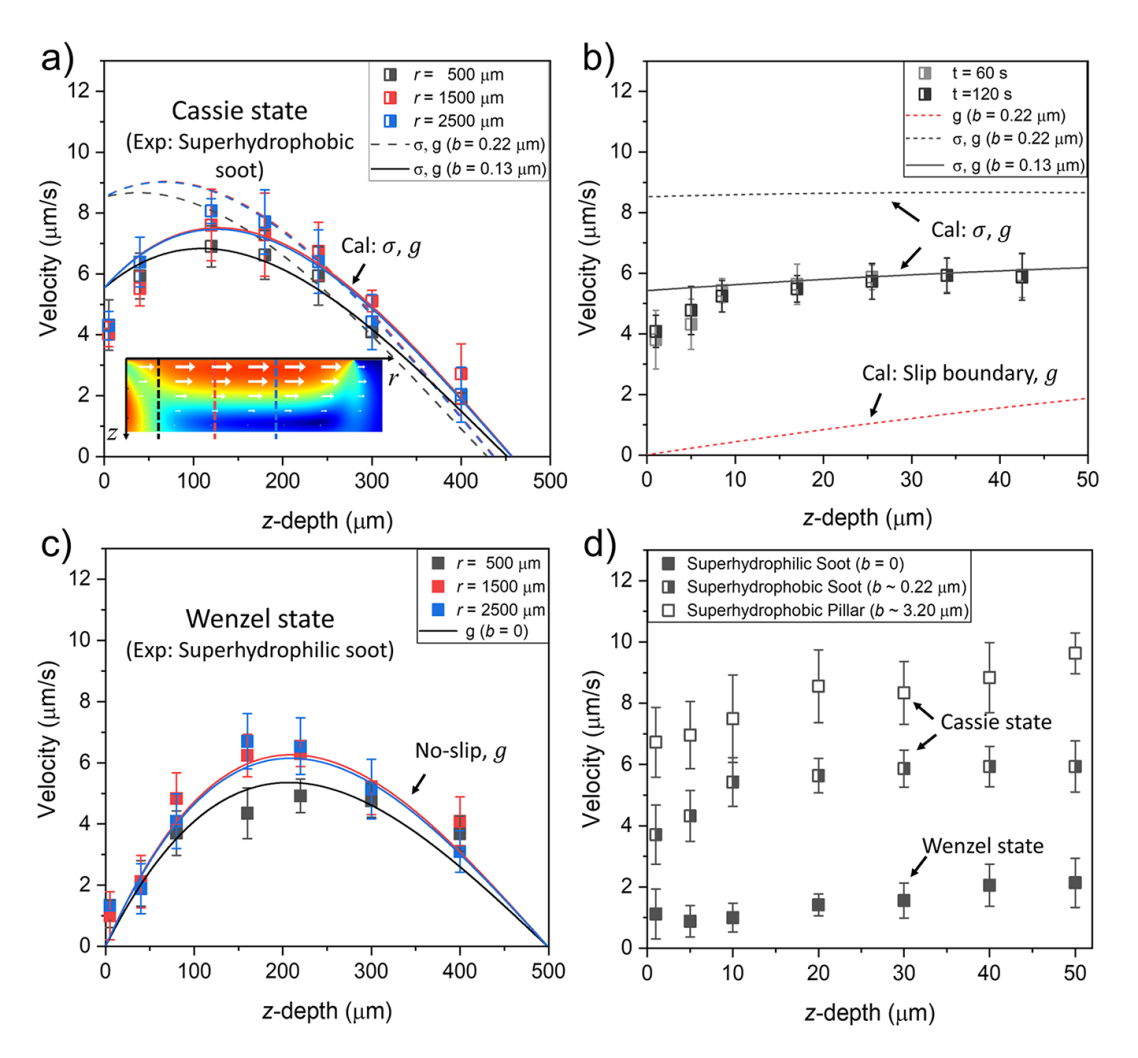
Photothermally induced velocity profiles in the liquid being in contact with solid substrates with different wettability and slip length. Symbols: measurements, Lines: calculations. (a) Cassie state on superhydrophobic surfaces. Experimental: Superhydrophobic soot surface, calculations: case 3 [Marangoni-stresses (“σ”) and buoyancy (“*g*”)]. Inset: simulated velocity magnitude. (b) Cassie state: close-up near the superhydrophobic soot surface for *r* = 500 μm. Experimental: superhydrophobic soot surface. Calculations: cases 2 (slip, *g*) and 3 (σ,*g*); (c) Wenzel state on superhydrophilic surface. Experimental: superhydrophilic soot surface. Calculation: no-slip boundary at the upper interface and buoyancy considered in the fluid (no-slip, *g*). Inset: simulated velocity magnitude; (d) comparison of the experimentally determined velocity profiles near the upper interface of the liquid layer (*z* < 50 μm; *r* = 500 μm).

To verify the mechanism of triggering Marangoni flows by photothermal stimuli, flow velocity measurements on surfaces with different wettability and morphology were performed: superhydrophobic soot (*b*_*l*__–soot_ ∼ 0.22 μm), superhydrophilic soot (*b*_hydrophilic_ = 0), and superhydrophobic pillar (*b*_*l*__–pillar_ ∼ 3.2 μm) surfaces. Here, superhydrophilic soot surfaces having the same surface properties and morphology as the superhydrophobic soot surfaces except for the wettability were tested first to compare the velocity distribution close to the surface and second to examine the thermally induced buoyancy effect when no Marangoni stresses are present. Superhydrophobic pillar surfaces were examined to study the role of surface morphology. Their slip length is supposedly 1 order of magnitude larger than the superhydrophobic soot surface. The corresponding experimentally determined velocity profiles in the liquid layer, are shown in Figure 4. They are measured perpendicular to the interface at *r* = 500, 1500, and 2500 μm from the center of the heated spot.

The experimentally determined velocity distribution on the superhydrophobic soot surface (Figure 4a) shows a mean velocity value *u* ∼ 4 μm/s close to the surface and increases with *z* until a maximum value *u*_max_ ∼ 8 μm/s appears around *z* ∼ 150 μm. Hence, the velocity profile does not correspond to the classic theoretical scenario where buoyancy is neglected and the maximum velocity appears directly at the surface (calculation case 1, Figure 4b). Rather, there is a basic agreement with case 3, considering both Marangoni stresses and buoyancy. The position of the maximum velocity depends on the choice of the effective slip length of the surface with the maximum being closer to the surface, the larger the slip length is. With the slip length estimated from the soot geometry of 0.22 μm, the calculated velocity profile shows a similar trend as the experimentally determined one. However, we observed an approximately factor of 2 larger velocity at the interface and correspondingly a velocity maximum closer to the interface. It has to be considered that first the boundary condition employed for the calculations is likely overestimating the boundary velocity due to Marangoni stresses, as discussed in the previous chapter. Second, there might be surface-active contaminations present at the air–water interface of the superhydrophobic surface. Contamination of the interface has been shown to effectively reduce slippage at the interface and would therefore also reduce the Marangoni velocity.^53,62−64^ Third, an estimation of the effective slip length of the randomly structured soot surface is per se not exact. All these effects may reduce the real value of the velocity at the interface. Hence with these considerations, the relation between the calculated velocity profile for a slip length of 0.22 μm and the experimental observation is reasonable. By comparing the shapes of the velocity profiles from the simulations with those from the experiments, we can determine an effective slip length of 0.13 μm in the simulations. This leads to a good agreement between calculations and experiments. Overall, this shows that indeed buoyancy plays an important role in our system and cannot a priori be neglected in such small-scale systems. A similar observation has been recently made by Li et al.^48^ for droplets.

Furthermore, we demonstrate that there is indeed a Marangoni flow present at the superhydrophobic surface and that there is not only buoyancy-driven motion. Figure 4b shows a close-up of the velocity profile near the superhydrophobic surface (*r* = 500 μm). There is a finite velocity at the water–air interface. Calculations considering buoyancy and a slip boundary condition with *b* = 0.22 μm but no Marangoni stresses (case 2) predict a much lower velocity at the interface. Hence, there must be Marangoni flow in the experiments. The same argument holds for the pillar surface (Figure S8).

In contrast, in control experiments with hydrophilic soot surfaces, pure buoyancy was observed (Figure 4c). Here, Marangoni stresses are absent due to the missing air–water interface. The velocity maximum is shifted away from the wall as compared to the superhydrophobic case, where the superposition of buoyancy and Marangoni effects moves the maximum closer to the surface.

In the experiments, we still detected a low velocity of ≈1 μm/s close to the surface. This velocity stems from the diffusion of the tracer particles, which have a random orientation. On the basis of the Stokes–Einstein relation,^65,66^ a diffusion velocity of the order of micrometer per second is to be expected (and in agreement with the observations). Overall, the agreement between the experimentally measured velocities and the calculations for pure buoyancy is very good. The velocity maximum is located at about *z* = 250 μm, has a similar magnitude, and the velocity directly at the hydrophilic surface tends to zero.

The dependence of the Marangoni velocity is shown in Figure 4d. From the no-slip scenario in the hydrophilic case, the Marangoni velocity increases with increasing slip length. The increase observed in experiments between the superhydrophobic soot and the superhydrophobic pillar surfaces is however not as strong as predicted by the calculations. A slip length of *b*_*l*__–pillar_ ∼ 3.2 μm leads to a predicted Marangoni velocity of 110 μm/s. This is a significantly larger overprediction as for the superhydrophobic soot. The same reasons for overprediction also apply here: the boundary condition itself as well as possible contamination of the air–water interface. Further reasons may lie in the different geometric designs of the interfaces, which might be prone to effects of surface contamination to different degrees.

After switching on the illumination, the flow first starts slowly and then stabilizes to a constant velocity after a time of about 60 s. This is exemplarily shown in Figure 4b for times *t* = 60s and *t* = 120 s after the start of the illumination. The flow development corresponds to the temperature response of the surface, which needs about 60 s to heat up to its final temperature (Figure S5). Therefore, all the experimental data were recorded after at least 60 s illumination when the flow became stable.

### Scaling Relations

To analyze the relation between thermocapillary and buoyancy flows, a natural approach is to compare the corresponding characteristic velocities. The Marangoni velocity for a superhydrophobic surface *u*_M_ scales as^12^. The magnitude of the buoyancy-driven velocity *u*_b_ is determined by a balance of buoyancy forces and viscous friction. The buoyancy force per unit volume is *F*_b_ = *βρ*_0_Δ*Tg*, where β is the coefficient of thermal expansion, ρ_0_ the initial density, and *g* the acceleration due to gravity. Δ*T* is the horizontal temperature difference at the upper liquid surface. Viscous forces scale as *F*_*v*_ ∼ *μU*/*L* with the characteristic velocity *U* and characteristic length scale *L*. In the present case, the latter corresponds to the height of the fluidic chamber *H*. Overall, balancing both contributions yields a characteristic buoyancy-driven velocity *u*_b_ ∼ *βρ*_0_*g*Δ*TH*^2^/μ. Hence,
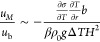
4With the typical values of our experiments and Δ*T* = 0.1 K, values of *u*_M_/*u*_b_ in the range of 0.05–0.7 are obtained, depending on the considered case (slip length and temperature gradient), indicating buoyancy being of somewhat larger influence than thermocapillary flow. Yet, the individual dependencies in eq 4 are delicate and, as will be discussed, need further investigation. Equation 4 is similar to the inverse of the dynamic Bond number *Bo* = *Ra*/*Ma*, which has been used to characterize the ratio of Marangoni to buoyancy flows at free water–air interfaces.^43,67,68^ Here, *Ra* is the Rayleigh number and *Ma* the Marangoni number. For free interfaces, the dynamic Bond number does not include a slip length, but the characteristic length *L*_*x*_ in the direction parallel to the interface at the same place. In the classical setups, this is both the fluid container size and the length over which the temperature gradient is applied.

Theoretically, increasing the slip length should also help in increasing *u*_M_/*u*_b_. Performing a series of numerical simulations with varying slip lengths and evaluating the typical velocities for *u*_M_ and *u*_b_ yields a velocity ratio *u*_M_/*u*_b_ that consistently with eq 4, increases linearly with the slip length *b* (Figure S9, at fixed thicknesses). Nevertheless, we did not find this linear dependency on the slip length in our experiments, even when considering that the temperature gradient was slightly smaller for the pillared surface. Further investigations are required to elucidate the role of surface geometry or contaminations as described above.

Furthermore, it has been observed for Marangoni flow at free interfaces that the ratio between thermocapillary flow and buoyancy experimentally rather follows an *L*_*x*_/*H*^3^-relation than a 1/*H*^2^-relation as in the reverse Bond number.^68^ Varying the height *H* in the numerical simulation similarly indicates a *b*/*H*^2^-relation. These observations are in line with recent findings for droplets indicating that the classical Bond number is not enough to characterize the interplay of thermocapillary and buoyancy.^48,69^

With respect to the question of how systems exploiting thermocapillary flow at superhydrophobic surfaces should be designed in order to take good advantage of thermocapillary flow, a computational variation of the system height illustrates the strong increase of buoyancy with the height *H* (Figure S10a). For the present superhydrophobic soot surface (*b* ∼ 0.1 μm), a desired dominance of the Marangoni flow over buoyancy would require system heights in the low μm regime. If larger slip lengths could be achieved, the critical thickness of the system could be raised to a few millimeters (Figure S10b).

Further conclusions can be drawn from the Marangoni number *Ma*, which characterizes the strength of Marangoni flow. The Marangoni number is a measure of the heat transport by convection due to surface tension gradients to the bulk heat transport by conduction and as such defined as *Ma* = *UL*/α, with α being the thermal diffusivity and *U* and *L* the characteristic velocity and length scale. In our case,  for a superhydrophobic surface and *L* = *H* for the convection. The Marangoni number for a superhydrophobic surface hence reads
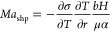
5This is, in principle, the same expression as derived by Yariv,^55,56^ only Yariv specifically considered longitudinal grooves with a pitch *p* that occurs instead of the slip length *b*. Since *b* ∼ *p*, both expressions are equivalent. For the present system, *Ma*_shp_ = 0.1–0.37. Hence, Marangoni flow is present, yet its strength is not extremely large. The Marangoni number also illustrates the influence of the heat conduction through the water, which was already observed experimentally. It could be further enhanced by increasing the slip length of the surface, increasing the temperature gradient or by employing omniphobic surfaces with different fluids of lower α.

## Conclusions

To manipulate liquid flow by light, we brought the liquid, in our case water, in contact with a superhydrophobic surface, in whose roughness features air is entrapped. The superhydrophobic surface is composed of soot to absorb the light efficiently and convert it to a temperature increase. A focused light is illuminating a spot on a superhydrophobic surface. This generates a nonuniform temperature distribution, which induces liquid flows. Even for a small temperature gradient, e.g., 0.2 K/mm generated on the solid surface, flows with a velocity of a few micrometers per second are created. Our experimental and numerical study has shown that it is indeed possible to create Marangoni flows on the superhydrophobic surface which drive the liquid. We have shown that the heating also induces buoyancy-driven flows, which have so far been neglected for superhydrophobic surfaces. Buoyancy-driven flows might act in the same direction as the Marangoni flows and are therefore not necessarily easy to distinguish. For the flow cell chosen, we have identified these two contributions and related their magnitudes with respect to each other.

This fundamental study helps to understand the mechanisms of fluid dynamics near superhydrophobic surfaces. From a practical view, optical actuation provides several advantages: on the one hand, by adjusting the light spot size and intensity, the temperature gradient on superhydrophobic surfaces can be easily controlled; on the other hand, the spatial adjustability of the light provides a flexibility in creating fluid motions at different positions. This could in future work allow one to achieve precise control of the thermocapillary flow on superhydrophobic surfaces via a simple illumination approach. Furthermore, a wide-field illumination, such as solar irradiation, could also be expected to trigger flows with this optothermal setup.

The design of the experimental setup plays a critical role, both on the macroscopic scale, i.e., with respect to the fluid reservoir of channel dimensions, as well as on the microscopic scale, i.e., with respect to the geometry and slip length of the superhydrophobic surface. We discussed the influencing factors in terms of scaling relations. This provides guidelines for designing thermocapillary-driven systems that best exploit Marangoni forces at superhydrophobic surfaces. Furthermore, buoyancy effects might also be exploited by a suitable design of the setup. With the presented optical triggering of buoyancy-driven flows, new propulsion methods for fluids could be developed.

## Supporting Information Available

Experimental details: materials, preparation of the soot-based superhydrophobic surface, preparation of the superhydrophobic surfaces with micro-pillar structures, measuring the flow velocity. Figures: schematic of the experimental chamber, schematic of the procedure for preparing PDMS-carbon black pillar structures on a glass substrate, SEM images of the modified micro-pillar structures, temperature distribution on a soot-based structure when irradiated by a blue laser from behind, experimental temperature evolution at the center of the irradiated spot on the soot-based surface as a function of the different laser powers, temperature distribution near the different upper surfaces, denomination of the boundaries in the simulation model, close up of the velocities in the liquid layer near the solid substrates with different wettability at *r* = 500 μm, numerically determined effect of liquid thickness on the velocity distribution in the liquid layer, numerically determined velocity ratio as a function of slip length, and boundary conditions for simulating thermocapillary-triggered flow in the setup (PDF)
