# Supplementary material for: Optical Manipulation of Liquids by Thermal Marangoni Flow along the Air–Water Interfaces of a Superhydrophobic Surface
Source: Langmuir. 2021 Jul 14;37(29):8677–86. doi: 10.1021/acs.langmuir.1c00539 (PMC8397335; doi:10.1021/acs.langmuir.1c00539)
Supplement: Supplementary file 1 — la1c00539_si_001.pdf [file la1c00539_si_001.pdf]

Supporting information

Optical manipulation of liquids by thermal Marangoni flow on a superhydrophobic surface

Aiting Gao,[†] Hans- Jürgen Butt[†], Werner Steffen[†], Clarissa Schönecker, [,†,‡]*

[†]Max Planck Institute for Polymer Research, Ackermannweg 10, D-55128, Mainz, Germany

[‡]TU Kaiserslautern, Group for Micro Fluid Mechanics, Gottlieb-Daimler-Straße 49, 67663 Kaiserslautern, Germany

* Correspondence and requests for materials should be addressed to C. S.
E-mail: schoenecker@mpip-mainz.mpg.de

Contents:

1. **Supplemental experiment details**

Materials
Preparation of the soot-based superhydrophobic surface
Preparation of the superhydrophobic surfaces with micro-pillar structures
Measuring the flow velocity (particle trajectories) in the liquid layer

2. **Supplemental Figures**

1. Supplemental experiment details

Materials

Tetraethyl orthosilicate (TEOS, 98 %), trichloro(1H, 1H, 2H, 2H-perfluorooctyl)silane (97 %) and Sodium dodecyl sulfate (SDS) were purchased from Sigma-Aldrich, Ethanol absolute, Hexane and iso-proponal and aqueous ammonia (25 %) were from Normapur.

Preparation of superhydrophilic soot surfaces

Glass slides were used as substrates. They were first ultrasonically cleaned in ethanol, acetone and isopropanol for 15 min, respectively, then treated with oxygen plasma (120 W, Diener Electronic Femto) for 10 min. The slides were then pre-coated with a thin layer of wax by rubbing a candle on the substrates back and forth several times (about 20 s). The pre-coated glass slide was placed close to the candle flame for about 5 s to melt the wax, then held above a candle flame for about 25 s. Thus, a thin layer of carbon nanoparticles (CNPs, diameter with 45 ± 5 nm) with an average thickness of ~ 30 μm was deposited on the slide.[1, 2] The soot layer was partly embedded in the wax coating, thus improving the adhesion between the soot and the glass surface. A following step-wise chemical silica deposition approach was done[3] to enhance the robustness of the deposited soot-based layer. A chemical vapor deposition step (CVD) was first performed by placing the soot-coated substrate in a closed desiccator for 48 h (with two vials, 2 mL ammonia and 2 mL TEOS, respectively), followed by an additional Stöber reaction by immersing the substrate in a liquid bath (mixed with 18 mL ethanol, 2 mL TEOS and 1.5 mL ammonia) for 4 h. Then the substrate was rinsed with ethanol. A thin SiO_2 layer that perfectly duplicated the soot layer's hierarchical structures was formed on the soot surface. Notably, the soot layer was not burnt away as it was essential to produce heat under light illumination due to its photo-thermal effect. Without further hydrophobic modification, the soot-based surface shows superhydrophilicity since the silica layer is hydrophilic in nature.

The superhydrophobic soot surface was obtained by further hydrophobic modification. It is done by placing the substrate in a desiccator (with a vial filled with 200 μL Trichloro(1H, 1H, 2H, 2H-perfluorooctyl)silane).The desiccator was sealed (vacuum the desiccator for about 30 s, when the pressure gauge showed < 200 mbar). After 3 h, the sample was taken out. The soot surface showed excellent superhydrophobicity ($\theta > 160$ °, $\theta_{CAH} < 5$ °).

Preparation of superhydrophobic pillar surfaces

The schema of the preparation of the superhydrophobic pillar surface is shown in **Figure S2**.

SU-8 pillar 3D structures (pillar diameter 10 μm, spacing 20 μm) were first built up on the glass substrate by photolithography. The SU-8 photoresist (10 μm) was first spin-coated onto a clean glass substrate, with a spin-coating speed of 1500 rpm/s, 30 s. A soft-baking procedure was then done by placing the substrate on a hot plate of 65 °C for 3 min, 85 °C for 10 min and followed by 65 °C for another 30 min, then cooled down to room temperature. Then, the SU-8 layer was exposed to UV light through a photomask for cross-linking and curing the film (with an exposure dose ~0.25 J/cm^2, 14 s). Afterwards, a hard-baking process was performed by placing the substrate on a hot plate at 65 °C for 5 min and 95 °C for 10 min, then cooled down to room temperature. In the following development procedure, the substrate was immersed in a commercial developer (50 mL, immersing in it for 2 min and followed by gently shaking for 1 min) and rinsed in isopropanol (slowly shaking) for 20 s. Then the substrate is dried with Nitrogen gas. Another post-baking procedure was done by placing the substrate on a heater at 152 °C for 20 min, to have a better appearance of the SU-8 patterns. A PDMS-carbon black mixture (mass weight ratio of 500:1; carbon black with the size around 25 ± 5 nm) was poured on the SU-8 mold surface and cured in an oven at 60 °C for 6 h. Then the PDMS-carbon black mixture layer could be easily peeled off from the mold. This process creates pillar structures (with a diameter a=10 μm, spacing d=20 μm and height h=30 μm) on the mixture layer. The cured PDMS-carbon black mixture layer was treated by an additional oxygen plasma treatment for 3 min before placing it onto a rigid glass substrate. This process allowed the PDMS layer to be more firmly attached to a glass substrate.

In order to enhance the stability of the Cassie-state of water on the pillared surface, a micro/nano-structured decoration was added by depositing a mono/multi-layer of micro-particles on the pillar surface.[221] First, the substrate with pillar structures was immersed in a MilliQ-water reservoir (~50 mL). Then the polystyrene particle dispersion (ϕ 1.1 μm, 1 wt% in ethanol) was dripped in the chamber (~ 2 mL). The micro-particles would either disperse in the bulk liquid or accumulate at the water-air interface. By dripping by solution (1 wt%, 2 to 3 μL) into the water reservoir, the particles assembled at the interface and were forced to pack together to form a closely-packed particle monolayer at the liquid-air interface. Then the water in the chamber was extracted through a needle at the bottom of the water reservoir. The closely-packed particle film approached the pillar

surface and is deposited there. With further hydrophobic modification, a superhydrophobic pillar surface was obtained ($\theta > 155°$, $\theta_{\mathrm{CAH}} \sim 15°$). An alternative way to modify micro/nano-structures on the pillar surfaces was done by directly spraying a commercial agent (soft 99, Glaco Mirror Coat 'Zero', containing nanoparticles and organic reagent) [4] on the pillar surface ($\theta > 150°$, $\theta_{\mathrm{CAH}} < 10°$).

Measuring the flow velocity (particle trajectories) in the liquid layer

Velocity measurements

The velocity profiles were obtained by tracking the moving tracers dispersed in the water using the MTrackJ plugin for ImageJ. First, for each velocity datapoint, a sequence of images (100 continuous images) obtained by confocal microscopy in *xyt* or *xytz* scanning mode were uploaded in ImageJ. The MTrackJ plugin was used to detect and manually track the moving particles in the successive frames via their pixel coordinates. Thereby, the particle trajectories were created. Then, individual particle velocities in the *xy* plane were obtained through the distance the particles traveled within the time elapsed between the corresponding frames. Finally, for each velocity datapoint, an average velocity value was calculated from the velocity values of at least 20 particles.

Flow pattern visualization (Fig. 2)

The mapping of the flow distribution in the fluorescent images was obtained through MATLAB (PIVlab plugin, 2018b, MathWorks) with the PIVlab code.[5, 6] After importing every 20 frames in the software, a ROI region (region of interest, with dimensions of width 480 pixels and height 480 pixels) was drawn in the first image and automatically applied to the following images. The images were pre-processed by selecting the contrast-limited adaptive histogram equalization (CLAHE, 20 pixels), high-pass pre-analysis filters (15 pixels) as well as the contrast stretch (0.01-0.32), to improve their resolutions. We selected the Fast Fourier transform window deformation (FFT) cross-correlation algorithm and defined the interrogation areas as a decreasing sequence of 240 × 120, 120 × 60 and 60 × 30 pixels to analyze the motions of the particles in the continuous image frames. We further selected velocity limits to filter outliers. The out of range velocity vectors were deleted and the missed vectors were replaced with values interpolated from nearest neighbors (orange colors). It should be noted that the interrogation area should not be too small (for example,

24 × 24 pixels), because the concentration of fluorescent particles in our system is relatively low (~1.2 $\times 10^6$/mL).

2. Supplemental Figures

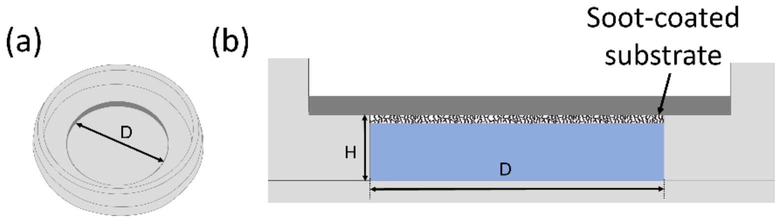

Figure S1. The schematic of the experimental chamber. (a) A commercial microscopy slide chamber was used with a hydrophilic bottom surface (reflective index: 1.52; thickness of bottom surface: 180 μm); (b) side view of the chamber. The prepared glass slide was placed on top of the chamber, which was filled with milli-Q water.

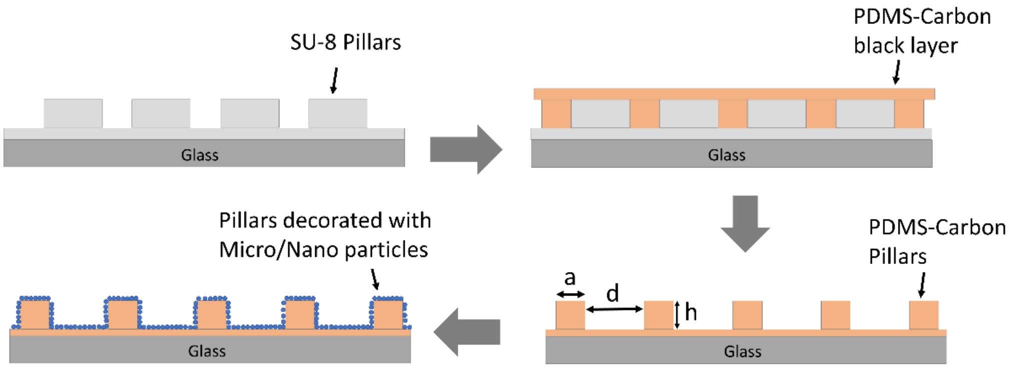

Figure S2. Procedure for preparing PDMS-carbon black pillar structures on a glass substrate, with the assistance of a SU-8 pillar surface.

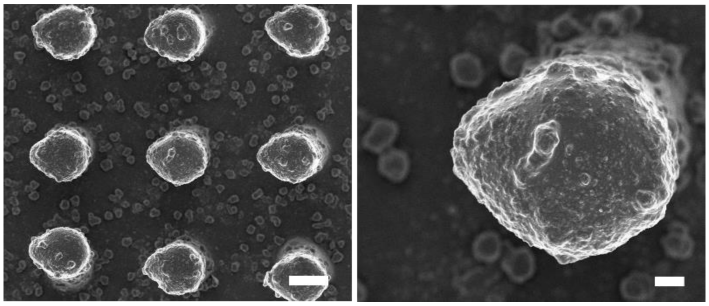

Figure S3. SEM images of the modified micro-pillar structures (Pillars with a diameter of *a* ~10 μm, spacing *b*~ 20 μm and height *h* ~30 μm). A commercial agent, soft 99 Glaco Mirror Coat ‘Zero’, containing nanoparticles was sprayed on the pillar surface. Scale bars: Left: 10 μm. Right: 2 μm.

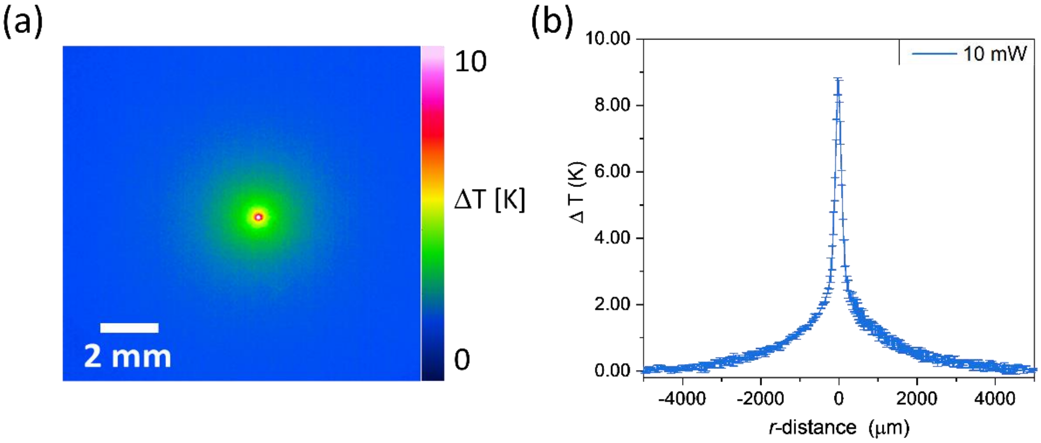

Figure S4. Temperature distribution on a soot-based surface when irradiated by a blue laser from behind (without water on the soot surface). (a) Infrared camera image of the soot surface under laser illumination and (b) the corresponding temperature distribution. (λ_{laser} = 488 nm, I_{laser} =10 mW, spot size diameter 350 μm, thickness of the soot layer: 25 ± 5 μm).

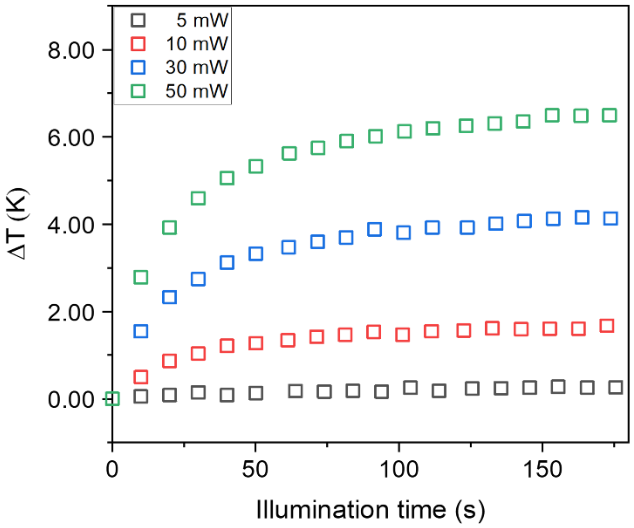

Figure S5. Experimental temperature evolution at the center of the irradiated spot ($r = 0$) on the soot-based surface as a function of different laser powers. (λ_{laser} = 488 nm, ϕ = 350 μm).

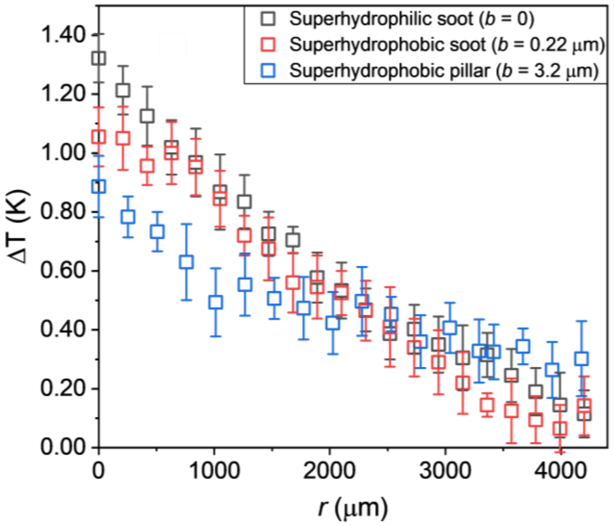

Figure S6. Temperature distribution near the different upper surfaces. The superhydrophobic and superhydrophilic surfaces have different slip lengths: $b \sim 0$ (Superhydrophilic soot surface), 10^{-7}m (Superhydrophobic soot surface) and 10^{-5} m (Superhydrophobic pillar surface). (λ_{laser} =488 nm, I_{laser} =10 mW, ϕ =350 μm).

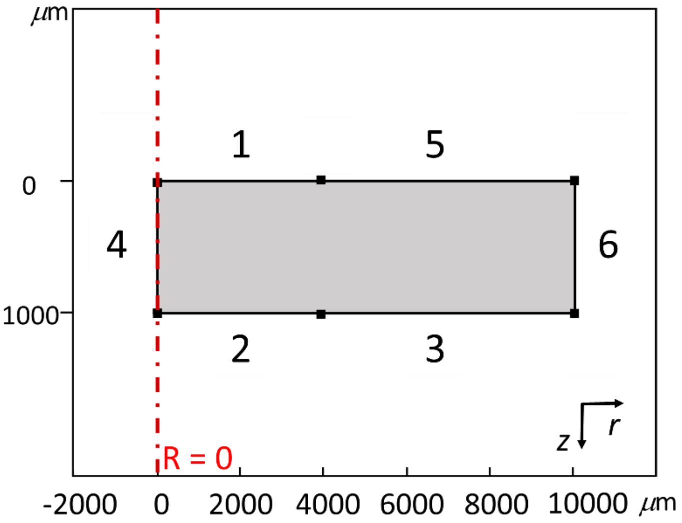

Figure S7. Denomination of the boundaries in the simulation model. Boundaries 1 to 6 are defined by different boundary conditions.

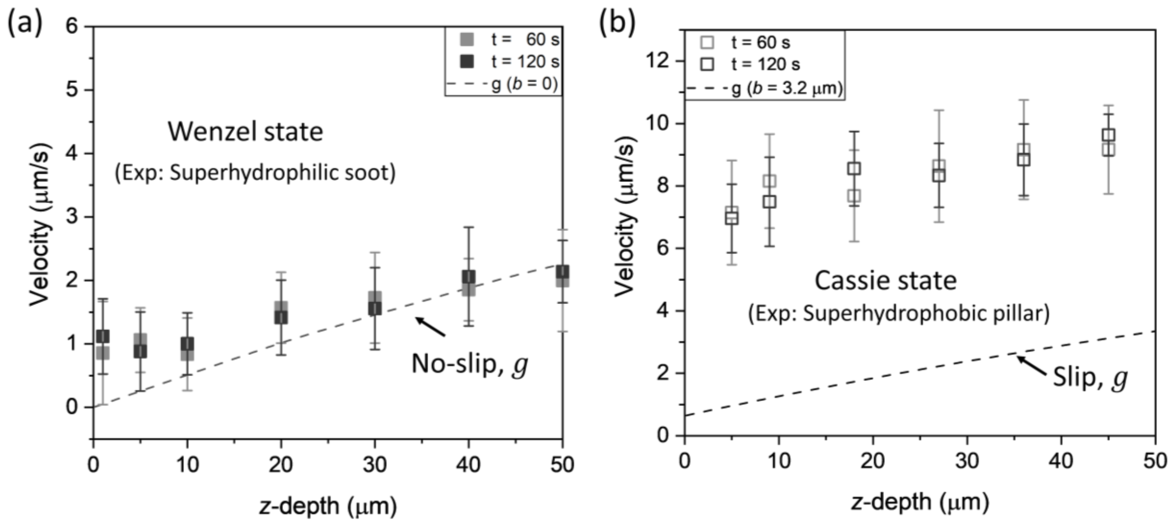

Figure S8. Close up of the velocities in the liquid layer near the solid substrates with different wettability at $r = 500$ μm. (a) Near the superhydrophilic soot surface ($b = 0$) Deviation of the experimental velocities from zero close to the surface is due to diffusion of the particles. (b) Near the superhydrophobic pillar surface ($b = 3.2$ μm). The numerical solution shown for comparison is case 2 without Marangoni forces. The larger velocities in the experiment show that there is Marangoni present. Scale bar: 50 μm.

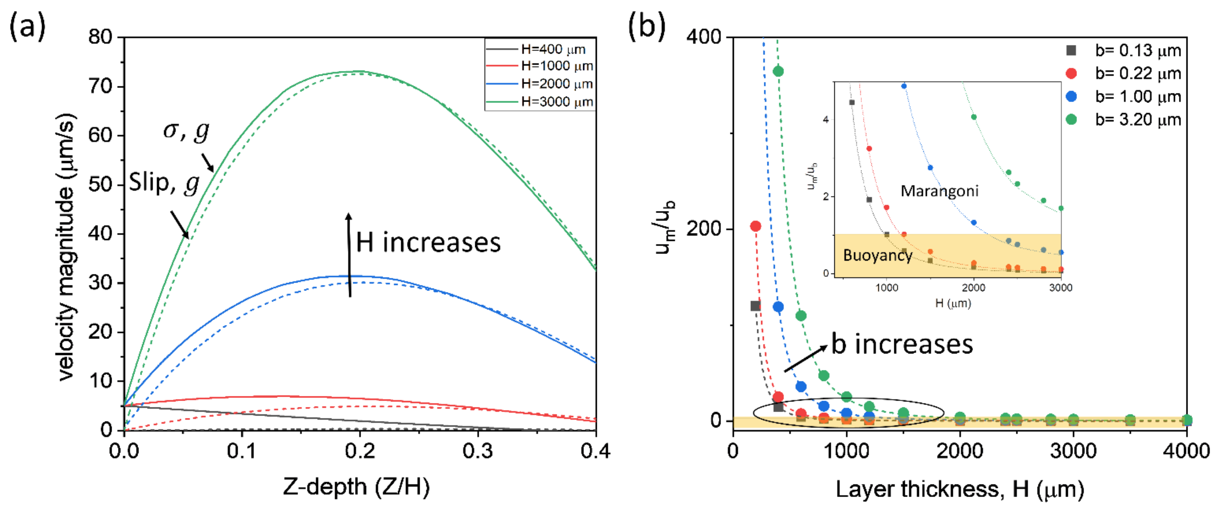

Figure S9. Numerically determined effect of liquid thickness on the velocity distribution in the liquid layer. (a) Horizontal velocity distribution in the liquid layer along z-direction for b =0.13 μm and $\frac{\partial \mathrm{T}}{\partial \mathrm{r}}$ =0.25 K/mm; Solid lines: Case 3 (σ, g); Dash lines: Case 2 (slip, g). (b) The velocity ratio ($\frac{u_M}{u_b}$) as a function of H for different slip length b. The orange shaded area highlights the buoyancy-dominated area ($\frac{u_M}{u_b}$ <1). The inset shows the magnified plots in the hollow ellipse area.

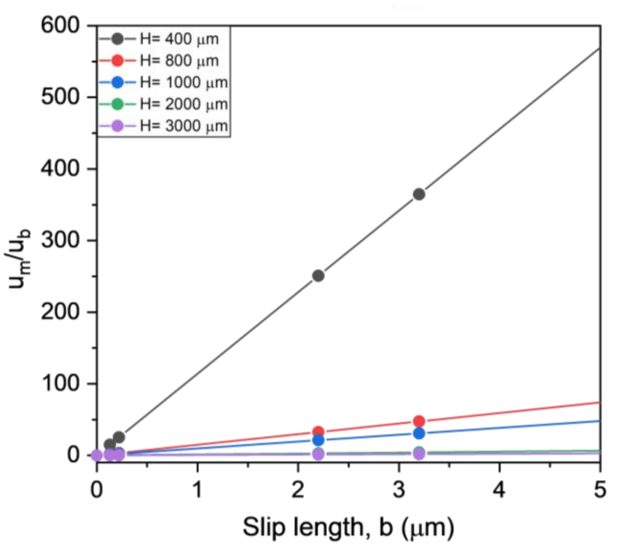

Figure S10. Numerically determined velocity ratio $\frac{u_M}{u_b}$ as a function of slip length b for $\frac{\partial T}{\partial r}$ =0.25 K/mm (in a liquid with a thickness H =1000 μm).

Table S1. Boundary conditions for simulating thermocapillary-triggered flow in the setup. The experimentally measured temperature distributions are used as an input on boundaries 1 and 2. They are slightly simplified as linear functions. Boundaries 3, 5 and 6 are no-slip walls with uniform ambient temperature $T_0 = 293.15\mathrm{K}$. Boundary 4 is the symmetry axis of the simulated axisymmetric geometry.

**Boundary**	**1**	**2**	**3, 5, 6**
Condition	Superhydrophobic surface	No-slip walls	
u	$u_r = -\frac{\partial\sigma}{\partial T}\left(\frac{\partial T}{\partial r}\right)\cdot b$; $u_z = 0$	$u_r = u_z = 0$	$u_r = u_z = 0$
T	$T_1 = T_0 + 1\mathrm{K} - r\left(\frac{\partial T}{\partial r}\right)$	$T_2 = T_0 + 0.9\mathrm{K} - r\left(\frac{\partial T}{\partial r}\right)\cdot 0.9$	$T = T_0$

Here, u is the flow velocity; u_r and u_z are the velocity component in the r-direction and z-direction, respectively; b is the corresponding slip length on the superhydrophobic surface.

References

1. Deng, X.; Mammen, L.; Butt, H. J.; Vollmer, D., Candle soot as a template for a transparent robust superamphiphobic coating. *Science* **2012**, *335*, 67-70.
2. Paven, M.; Fuchs, R.; Yakabe, T.; Vollmer, D.; Kappl, M.; Itakura, A. N.; Butt, H. J., Mechanical Properties of Highly Porous Super Liquid-Repellent Surfaces. *Adv. Funct. Mater.* **2016**, *26*, 4914-4922.
3. Stöber, W.; Fink, A.; Bohn, E., Controlled growth of monodisperse silica spheres in the micron size range. *J. Colloid Interface Sci.* **1968**, *26*, 62-69.
4. Vakarelski, I. U.; Patankar, N. A.; Marston, J. O.; Chan, D. Y.; Thoroddsen, S. T., Stabilization of Leidenfrost vapour layer by textured superhydrophobic surfaces. *Nature* **2012**, *489*, 274-7.
5. Thielicke, W.; Stamhuis, E. J., PIVlab – Towards User-friendly, Affordable and Accurate Digital Particle Image Velocimetry in MATLAB. *Journal of Open Research Software* **2014**, *2*.
6. Thielicke, W. The flapping flight of birds: Analysis and application. University of Groningen, 2014.
